# Denise Assunta Coia (Macdonald), DBE, FRCPsych, FRCPSG, FRSE

**DOI:** 10.1192/bjb.2020.86

**Published:** 2021-04

**Authors:** Alexander L. Macdonald, Andrew C. Macdonald

**Formerly Consultant Psychiatrist and Principal Medical Officer to the Scottish Government**

**Figure:**
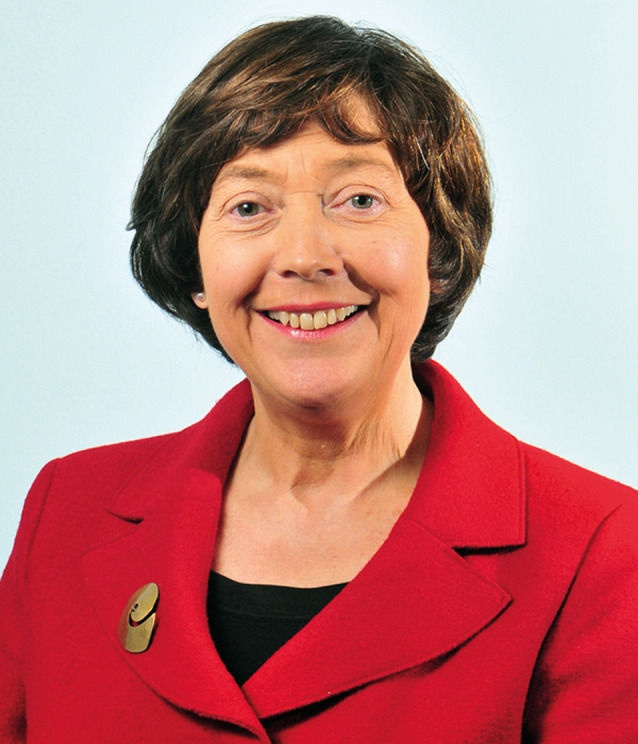

Denise Coia, who died peacefully on 9 April 2020 at the age of 67 after a short illness, was a leading Scottish psychiatrist with an international reputation. From 2011, on its foundation, she was the first Chair of Healthcare Improvement Scotland (HIS). In this capacity, with great tenacity, she skilfully built a strong and internationally respected organisation that drove through improvements in the quality of healthcare in Scotland. She was courageous enough to assert the organisation's independence from government and Ministers, through the publication of some hard-hitting hospital inspection reports.

She found interesting metaphors to express her thoughts and was often open and candid in doing so. Fearing that, without reform, the National Health Service (NHS) could consume infinitely more resources, she took the opportunity – in an HIS public annual review meeting with the government – to give Ministers a warning about a possible parallel with the fate of the world's only seven-masted schooner, the *Thomas W. Lawson*. In simply adding more masts to compete with steamships, the schooner had not kept pace with the revolution in maritime trade. Ultimately, it failed, sinking in a storm. Denise's message was abundantly clear – there was a need for the NHS to redesign and change with the times to survive.

Throughout her career, and especially in her later years, she championed the mental health of children and young people. Denise passionately believed in investing more in child and adolescent mental health services. She was therefore a natural choice to Chair the Scottish Government/Convention of Scottish Local Authorities (COSLA) Taskforce on Children and Young People's Mental Health. In her last few years, she also served as Convenor of Children in Scotland. In her typically energetic and inclusive way, she initiated a huge listening exercise across Scotland and relished the chance to talk to hundreds of people but most importantly children, young people and families. Her response when hearing excuses about service failures was that she was listening to what children and young people were telling her and, under her watch, the failures they had experienced would not be repeated.

Earlier in her career as consultant psychiatrist at the Florence St Day Hospital and Leverndale Hospital, Glasgow, where she served from 1987 to 2011, she was largely based in the Gorbals, one of the most deprived communities in Glasgow. It was there that she developed her life-long commitment to improving the mental health of the most vulnerable and disadvantaged in society. She was one of the pioneers of specialist community mental health services across Glasgow. From 1998 to 2006, she served as part-time Mental Health Advisor to Greater Glasgow Health Board. Then, on secondment from her consultant post, from 2006 until her retirement in 2011, she was part-time Principal Medical Officer Mental Health to the Scottish Government. She was an honorary senior lecturer in the Department of Psychiatry in the University of Glasgow.

Denise took considerable interest in medical education and in supporting the development and training of junior doctors. She chaired the General Medical Council (GMC) Quality Scrutiny Group overseeing the quality of postgraduate and undergraduate medical training in the UK. With Professor Michael West, she also led an extensive review into the mental well-being of medical students and doctors, which was published in 2019 under the title *Caring for Doctors, Caring for Patients*.

An only child, Denise Coia was born in Glasgow on 4 June 1952. Her father Joe and her mother Jill (née Dummer) ran a local fish and chip shop in Milngavie, where she was brought up. She attended Glasgow's Notre Dame High School and subsequently won a place to study medicine at the University of Glasgow. She graduated in 1976 and initially trained for a career in obstetrics, but finding this ‘boring’, she switched to train in clinical psychiatry. She trained in the South Glasgow registrar training scheme based at Leverndale & Southern General Hospital, followed by senior registrar training in the West of Scotland scheme.

Denise had a passionate interest in art. In Glasgow's European City of Culture year in 1990 she established an exhibition from her mental health centre base of artwork by those who had suffered mental ill health and their carers.

Among many official positions, Denise served as Vice President of the Royal College of Psychiatrists UK and Chair of the Royal College of Psychiatrists in Scotland. She was in receipt of many public honours, being appointed Dame Commander of the Order of the British Empire in 2016. She was elected a Fellow of the Royal Society of Edinburgh in 2018.

Even with all her achievements, Denise remained firmly down to earth. She brought *joie de vivre*, laughter and a wonderful sense of humour – always with a sparkle and not without a hint of mischief and self-deprecation. Denise drew admiration and respect from all quarters for her personal courage and straight talking. She had a formidable intellect and enjoyed robust debate but was always ready to see the other side of a well-argued case. She loved socialising and catching up across her wide network of friends and colleagues – and particularly enjoyed exchanging news. This was still evident even in the weeks before her health finally deteriorated – she was always planning the next coffee or lunch. She had many outside interests, including tennis, art, horse riding, skiing and reading.

She met her future husband Archie Macdonald, originally from Benbecula, a marine engineer with Caledonian MacBrayne, at Glasgow University. They married in 1977. She was immensely proud of their two sons, Alexander, a paediatric surgeon in London, and Andrew, an interventional radiologist in Oxford.

Denise leaves behind her beloved husband Archie, her sons Alexander and Andrew and three grandchildren.

